# Insulin-Like Growth Factor-1 and Bone Morphogenetic Protein-2 Jointly Mediate Prostaglandin E2-Induced Adipogenic Differentiation of Rat Tendon Stem Cells

**DOI:** 10.1371/journal.pone.0085469

**Published:** 2014-01-09

**Authors:** Junpeng Liu, Lei Chen, You zhou, Xiangzhou Liu, Kanglai Tang

**Affiliations:** 1 Department of Orthopaedics, Southwest Hospital, Third Military Medical University, Chongqing, China; 2 Department of Orthopaedics, Wuhan General Hospital of Guangzhou Military Region, Wuhan, China; Cardiological Center Monzino, Italy

## Abstract

Tendinopathy is characterized histopathologically by lipid accumulation and tissue calcification. Adipogenic and osteogenic differentiation of tendon stem cells (TSCs) are believed to play key roles in these processes. The major inflammatory mediator prostaglandin E2 (PGE2) has been shown to induce osteogenic differentiation of TSCs via bone morphogenetic protein-2 (BMP-2), and BMP-2 has also been implicated in adipogenic differentiation of stem cells. We therefore examined the mechanisms responsible for PGE2-induced adipogenesis in rat TSCs *in vitro*. Insulin-like growth factor-1 (IGF-1) mRNA and protein were significantly up-regulated in PGE2-stimulated TSCs, measured by quantitative reverse transcription-polymerase chain reaction and enzyme-linked immunosorbent assay, respectively. Incubation with specific inhibitors of cAMP, cAMP-dependent protein kinase A (PKA), and CCAAT/enhancer binding protein-δ (CEBPδ) demonstrated that IGF-1 up-regulation occurred via a cAMP/PKA/CEBPδ pathway. Furthermore, neither IGF-1 nor BMP-2 alone was able to mediate adipogenic differentiation of TSCs, but IGF-1 together with BMP-2 significantly increased adipogenesis, indicated by Oil Red O staining. Moreover, knock-down of endogenous IGF-1 and BMP2 abolished PGE2-induced adipogenic differentiation. Phosphorylation of CREB and Smad by IGF-1 and BMP-2, respectively, were required for induction of the adipogenesis-related peroxisome proliferator-activated receptor γ2 (PPARγ2) gene and for adipogenic differentiation. In conclusion, IGF-1 and BMP-2 together mediate PGE2-induced adipogenic differentiation of TSCs *in vitro* via a CREB- and Smad-dependent mechanism. This improved understanding of the mechanisms responsible for tendinopathies may help the development of more effective therapies.

## Introduction

Tendons are constantly subjected to stress and mechanical loading, which can lead not only to acute tendon injuries, but also to chronic degenerative tendinopathies [1]. The Achilles, patella, elements of the rotator cuff, forearm extensors, biceps brachi and tibialis posterior tendons are most vulnerable to tendinopathies [2], which are a common clinical problem in both athletes and the general public. They involve degenerative changes exacerbated by overuse and mechanical loading [2], and are characterized histopathologically by lipid accumulation and tissue calcification [3], [4], [5], [6].

The presence of cells with multilineage differentiation potential, termed tendon stem cells (TSCs), has been demonstrated in humans [7], mice [7], [8], rabbits [9] and rats [10]. TSCs can differentiate into non-tenocyte lineages such as adipocytes, chondrocytes and osteocytes under suitable conditions [7], [9], [10], [11], [12], [13], providing a possible mechanism for the osteogenic and adipogenic changes associated with tendinopathies.

PGE2 is a major mediator of pain and acute inflammation [14]. Mechanical stretching of tendon fibroblasts (tenocytes) or tendon explants has been shown to increase the production of PGE2 in *in vitro* studies [15], [16], [17], [18], [19], [20]. PGE2 treatment may result in degenerative changes of the tendon characterized by lipid accumulation and tissue calcification, partly by inducing the differentiation of TSCs into non-tenocytes, including adipocytes and osteocytes [9], [11], [21].

We previously demonstrated that PGE2 induced BMP-2 production through phosphoinositide 3-kinase (PI3K)-Akt signalling [21], and BMP-2 has been shown to play a role in tendon calcification [22] and to mediate PGE2-induced osteogenic differentiation in TSCs [23]. Huang et al. found that the BMP signalling pathway was also required for commitment of C3H10T1/2 pluripotent stem cells to the adipocyte lineage [24]. However, the role of BMP-2 in the adipogenic differentiation of TSCs remains unclear. Insulin-like growth factor 1 (IGF-1) is also known to promote adipogenic differentiation [25], [26], and was increased in tendons subjected to repetitive mechanical loading *in vivo* [26].

In this study, we investigated the roles of IGF-1 and BMP-2 in PGE2-induced adipogenic differentiation of cultured rat TSCs, and defined the roles of downstream cAMP response element-binding protein (CREB) and Smad signaling in the effects of IGF-1 and BMP-2. The findings of this study will help to clarify the nature of tendinopathies and so help in the development of future therapeutic strategies for these chronic conditions.

## Materials and Methods

### Isolation and culture of rat TSCs

All experiments were approved by the Animal Research Ethics Committee, Third Military Medical University, China. Rat TSCs were isolated from Sprague-Dawley rats and cultured, as described previously [21]. To determine if PGE2 induced adipogenic differentiation, TSCs were seeded in six-well plates at a density of 6×10^4^/well and were incubated with 0, 10, 50, 100 or 200 ng/ml PGE2 (Sigma-Aldrich, St. Louis, MO, USA) for 7 days, or with PGE2 100 ng/ml for 0, 3, 7 or 10 days. To determine the ability of BMP-2 to induce adipogenic differentiation, rat TSCs were incubated with 0, 10, 50, 100 or 200 ng/ml BMP-2 (Sigma-Aldrich) for 7 days, or with BMP-2 (100 ng/ml) for 0, 3, 7 or 10 days. The effects of IGF-1 on adipogenic differentiation of rat TSCs were determined by incubation with IGF-1 (Sigma-Aldrich) alone (10 nM), or with 0, 1, 5, 10 or 20 nM IGF-1 in combination with 100 ng/ml BMP-2. The cAMP synthesis inhibitor 2′,5′-dideoxyadenosine (ddA, 10 μmol/l, Sigma-Aldrich) and the PKA inhibitor H-89 (10 μM, Sigma-Aldrich) were added to PGE2-stimulated TSCs to determine the role of the cAMP/PKA/CEBPδ pathway.

### RNA interference

Short-hairpin (shRNA) lentiviral particles targeting BMP-2, IGF-1, CEBPδ, CREB and Smad1 were purchased from Santa Cruz Biotechnology (Santa Cruz, CA, USA) and used to transduct cells, according to the manufacturer's instructions.

### Oil Red O Staining

Adipocytes were identified by staining with Oil Red O (Sigma-Aldrich), as described previously [27]. The area of the cells stained with Oil Red O was measured by ImagePro Plus software (Palmerton).

### RNA isolation and quantitative real-time polymerase chain reaction (qRT-PCR)

mRNA expression levels of IGF-1 and peroxisome proliferator-activated receptor γ (PPARγ) were determined by qRT-PCR. Total RNA was extracted from cells using TRIzol reagent following the protocol provided by the manufacturer (Invitrogen). cDNA was synthesized from total RNA using a Superscript III first-strand synthesis kit (Invitrogen). qRT-PCR was performed using a SYBR Green RT-PCR kit (Qiagen, Hilden, Germany) and an ABI Prism 7900 Sequence Detection System (PE Applied Biosystems, Foster City, CA, USA). Expression levels were calculated relative to expression of the housekeeping gene glyceraldehyde 3-phosphate dehydrogenase (GAPDH). The primer sequences used in this research are listed in Table 1.

**Table 1:** Primers for PCR.

Gene	Primer
PPARγ	F: 5′- ATGACCACTCCCATTCCTTT -3′
	R: 5′- TGATCGCACTTTGGTATTCTT -3′
PPARγ2	F: 5′-CCCTTTACCACGGTTGATTTCTC-3′
	R: 5′- GCAGGCTCTACTTTGATCGCACT-3′
IGF-1	F: 5′- GGCATTGTGGATGAGTGTTG-3′
	R: 5′- GCTGGGACTTCTGAGTCTTGG-3′
GAPDH	F: 5′-GGCAAGTTCAACGGCACAG-3′
	R: 5′- CGCCAGTAGACTCCACGAC-3′

F = forward; R = reverse; GAPDH = glyceraldehyde phosphate-3 dehydrogenase (internal control).

### Measurement of IGF-1 and cAMP levels

IGF-1 protein levels were measured using a mouse IGF-1 enzyme-linked immunosorbent assay (ELISA) kit (EMI1001-1; AssayPro, Saint Charles, MO, USA). Intracellular cAMP levels were measured using a cAMP enzyme immunoassay (EIA) kit (Enzo Life Sciences, Lörrach, Germany) according to the manufacturers' protocols.

### Protein extraction and western blotting

Cells were rinsed, harvested, and lysed in hypotonic buffer A (10 mM Hepes, 10 mM KCl, 2 mM MgCl_2_, 500 μM DTT, 1mM Na_3_VO_4_, 10 mM NaF, 1% Triton X-100) containing a mixture of protease inhibitors (Thermo Fisher Scientific Inc., Rockford, IL, USA). Lysates were centrifuged at 14000 rpm for 15 min and supernatants were collected as the cytosolic fraction. Pellets were resuspended in hypertonic buffer B (25% glycerol, 420 μM NaCl, 200 μM Na_2_EDTA, 500 μM DTT, 1 mM Na_3_VO_4_, 10 mM NaF, and phosphatase inhibitors) and shaken on ice for 30 min. Lysates were centrifuged at 12000 rpm for 5 min and the supernatants (nuclear extracts) were diluted in buffer C (20 mM Hepes, 20% glycerol, 50 mM KCl, 200 μM EDTA, 500 μM DTT, 1 mM PMSF). Protein concentrations were measured using a BCA protein assay kit (Thermo Fisher Scientific Inc.). Western blotting was carried out as described by Ji et al. [28], using the following antibodies: rabbit polyclonal anti-PKA C-α, anti-CREB, and anti-phosphorylated CREB (p-CREB) (Ser133), (all from Cell Signaling Technologies, Beverly, MA, USA); rabbit polyclonal anti-p-PKA IIα reg (Ser 96)-R, anti-CEBPδ, anti-pSmad1/5/8, anti-Smad, (all from Santa Cruz Biotechnology). Antibodies to β-actin and histone deacetylase (HDAC) (Santa Cruz Biotechnology) were used as loading controls. Results were visualized and images captured using a LiCoR Odyssey imager (LI-COR Biosciences, Lincoln, NE, USA).

### Statistical analysis

Data were expressed as mean ± SD. Multiple comparisons were made using one-way analysis of variance followed by Fisher's tests. A P value <0.05 was considered to be statistically significant.

## Results

### PGE2 induces adipogenic differentiation

**Figure 1:**
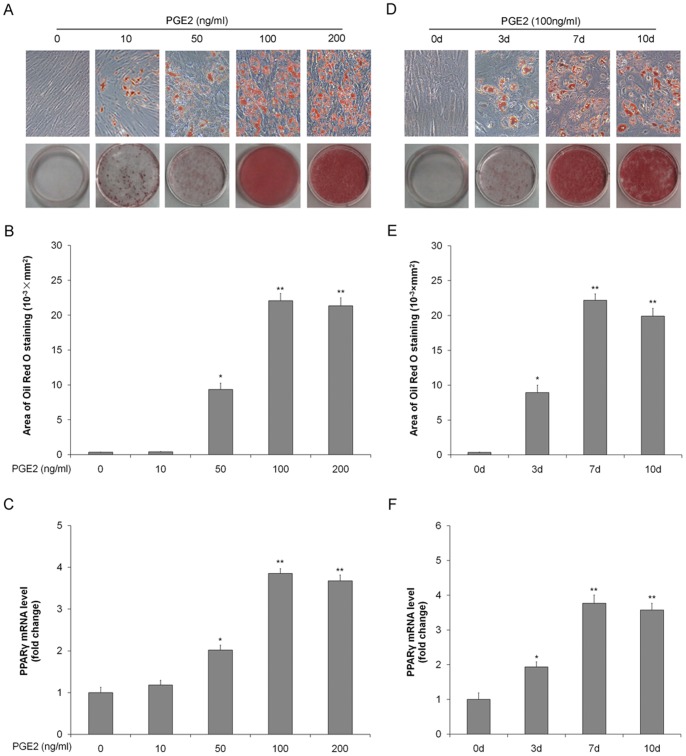
PGE2-induces adipogenic differentiation in rat TSCs. Rat TSCs were incubated with 0, 10, 50, 100, or 200/ml PGE2 for 7 days. (A) Adipogenic differentiation detected by Oil Red O staining; (B) the Oil Red O staining areas in the cells were assessed as described in the text; (C) PPARγ mRNA levels determined by qRT-PCR. Rat TSCs were incubated with 100 ng/ml PGE2 for 0, 3, 7, or 10 days. (D) Adipogenic differentiation detected by Oil Red O staining; (E) the areas stained with Oil Red O in the cells were assessed; (F) PPARγ mRNA levels determined by qRT-PCR. The mRNA levels were normalized using GAPDH. Results represent the mean ± SD. *P<0.05, **P<0.01 with respect to TSCs without PGE2.

To define the role of PGE2 in adipogenesis, rat TSCs were incubated with increasing doses of PGE2 for 7 d, or with 100 ng/ml PGE2 for different time. The presence of adipocytes was assessed by Oil Red O staining and mRNA expression of the adipogenic gene PPARγ. As shown in Fig. 1, adipocyte numbers and PPARγ expression were increased after PGE2 treatment in time- and dose-dependent manners, levelling off at a concentration of 100 ng/ml and duration of 7 days, respectively. These results indicated that PGE2 induced adipogenic differentiation of TSCs.

### BMP-2 alone does not mediate PGE2-induced adipogenic differentiation

**Figure 2:**
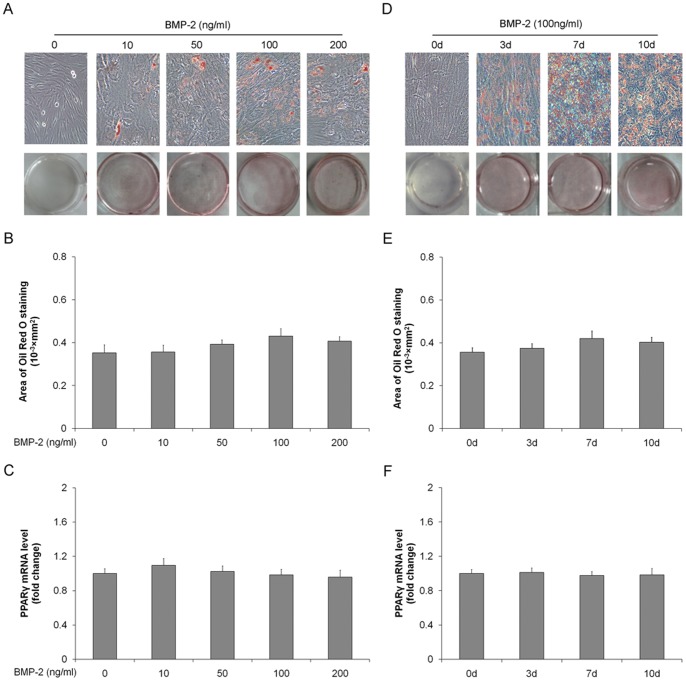
BMP-2 alone does not induce adipogenic differentiation in rat TSCs. TSCs were incubated with (0, 10, 50, 100, or 200 ng/ml BMP-2 for 7 days. (A) Adipogenic differentiation detected by Oil Red O staining; (B) the areas stained with Oil Red O in the cells were assessed; (C) PPARγ mRNA levels determined by qRT-PCR. TSCs were incubated with 100 ng/ml BMP-2 for 0, 3, 7, or 10 days. (D) Adipogenic differentiation detected by Oil Red O staining; (E) the Oil Red O staining areas in the cells were assessed; (F) PPARγ mRNA levels determined by qRT-PCR. The mRNA levels were normalized using GAPDH. Results represent the mean ± SD.

To determine if BMP was able to mediate PGE2-induced adipogenic differentiation directly, we assessed the differentiation of TSCs incubated with increasing concentrations of BMP-2, or with 100 ng/ml BMP-2 for the indicated days. BMP-2 had no significant effect on adipocyte number or on PPARγ expression (Fig. 2). This indicated that BMP-2 alone was unable to mediate PGE2-induced adipogenic differentiation, and suggested that additional factors are required.

### PGE2 upregulates IGF-1 via the cAMP/PKA/CEBPδ pathway

**Figure 3:**
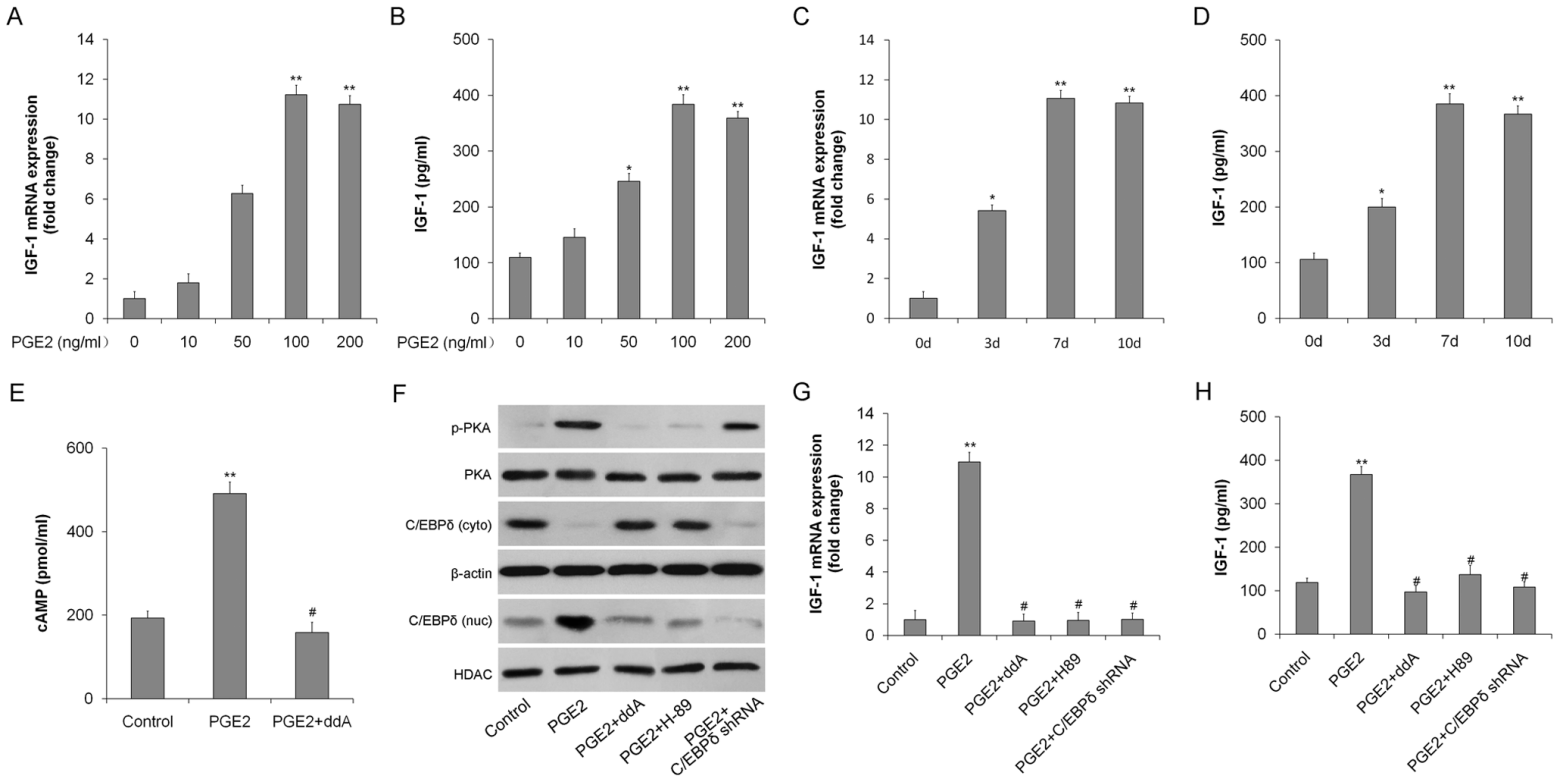
IGF-1 expression in rat TSCs is upregulated by PGE2 via the cAMP/PKA/CEBPδ pathway. TSCs were incubated with 0, 10, 50, 100, or 200/ml PGE2 for 7 days. (A) IGF-1 mRNA expression was determined by qRT-PCR; (B) IGF-1 protein levels were quantitated by ELISA. TSCs were incubated with 100 ng/ml PGE2 for 0, 3, 7, or 10 days. (C) IGF-1 mRNA expression was determined by qRT-PCR; (D) IGF-1 protein levels were quantitated by ELISA. (E) TSCs were incubated in medium containing PGE2 (100 ng/ml), with or without the cAMP inhibitor ddA (10 μmol/l) and intracellular cAMP levels were determined by EIA. TSCs were incubated in medium containing PGE2 (100 ng/ml) with or without the cAMP inhibitor ddA (10 μmol/l), the PKA inhibitor H-89 (10 μM), or after transduction with the CEBPδ shRNA. (F) p-PKA and total PKA, nuclear (nuc) and cytoplasmic (cyto) CEBPδ protein levels determined by western blotting. HDAC (nuc) and β-actin (cyto) were used as loading controls; (G) IGF-1 mRNA levels determined by qRT-PCR. The mRNA levels were normalized using GAPDH; (H) IGF-1 protein levels determined by ELISA. Results represent the mean ± SD. *P<0.05, **P<0.01 with respect to TSCs without PGE2; #P<0.05 with respect to TSCs with PGE2.

Rat TSCs were incubated with PGE2 (0–200 ng/ml) for 7 days to induce adipogenic differentiation. IGF-1 mRNA and protein levels, measured by qRT-PCR and ELISA, respectively, increased in dose-dependent manners in response to PGE2 treatment (Fig. 3A and B). PGE2 also upregulated IGF-1 mRNA expression and protein synthesis in a time-dependent manner (Fig. 3C and D).

To determine if PGE2 induced IGF-1 secretion via a cAMP/PKA/CEBPδ pathway, TSCs were incubated in medium containing PGE2 (100 ng/ml) for 7 days. PGE2 increased intracellular cAMP in TSCs (Fig. 3E), activated cAMP-dependent protein kinase PKA, and stimulated nuclear translocation of CEBPδ (Fig. 3F). Addition of the cAMP synthesis inhibitor ddA, the PKA inhibitor H-89, or CEBPδ shRNA markedly inhibited PGE2-induced IGF-1 production at both mRNA and protein levels (Fig. 3G and H), indicating that PGE2 stimulated IGF-1 expression through cAMP/PKA/CEBPδ pathway.

### BMP2 and IGF-1 together mediate PGE2-induced adipogenic differentiation

**Figure 4:**
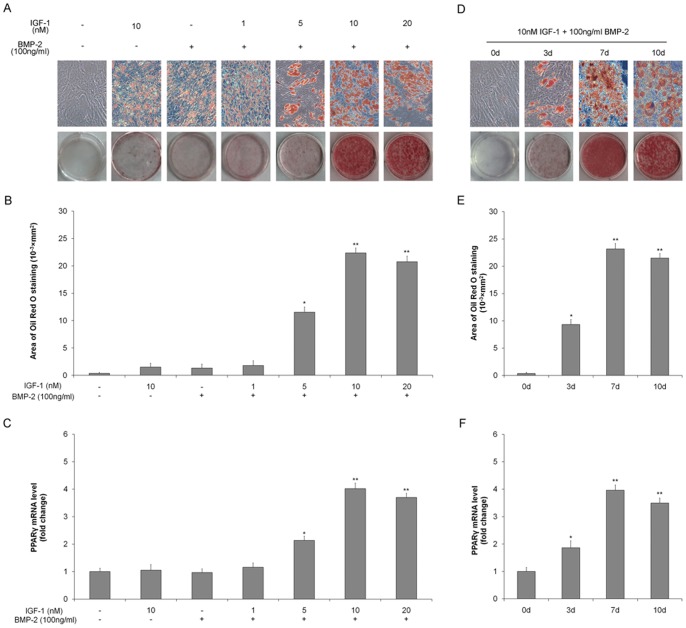
IGF-1 and BMP-2 induce adipogenic differentiation of rat TSCs. TSCs were incubated with 0, 1, 5, 10, or 20-1 and 100 ng/ml BMP-2 for 7 days. (A) Adipogenic differentiation detected by Oil Red O staining; (B) the Oil Red O staining areas in the cells were assessed; (C) PPARγ mRNA levels determined by qRT-PCR. TSCs were incubated with 10 nM IGF-1 plus 100 ng/ml BMP-2 for 0, 3, 7, or 10 days. (D) Adipogenic differentiation detected by Oil Red O staining; (E) the Oil Red O staining areas in the cells were assessed; (F) PPARγ mRNA levels determined by qRT-PCR. The mRNA levels were normalized using GAPDH. Results represent the mean±SD. *P<0.05, **P<0.01 with respect to TSCs without PGE2.

**Figure 5:**
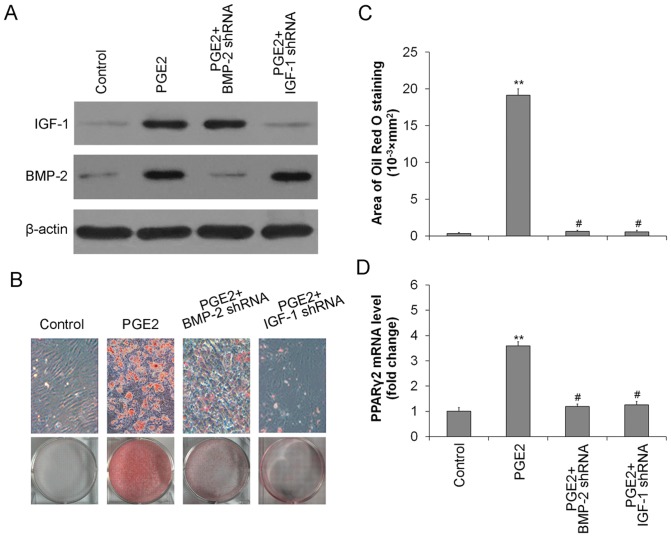
Knock down endogenous IGF-1 and BMP2 abolished PGE2-induced adipogenic differentiation. Rat TSCs were treated with PGE2 (100 ng/ml) with or without BMP-2 shRNA or IGF-1 shRNA. (A) Levels of IGF-1 and BMP2 were determined by western blotting. β-actin was used as loading controls. (B) Adipogenic differentiation detected by Oil Red O staining; (C) The Oil Red O staining areas in the cells were assessed; (D) PPARγ mRNA levels determined by qRT-PCR. The mRNA levels were normalized using GAPDH. Results represent the mean ± SD. **P<0.01 with respect to TSCs without PGE2; #P<0.05 with respect to TSCs with PGE2.

IGF-1 in the absence of BMP-2 failed to induce adipogenic differentiation in TSCs. However, IGF-1 together with BMP-2 significantly induced adipogenic differentiation in TSCs, as demonstrated by Oil Red O staining (Fig. 4A). Both adipocyte numbers and PPARγ mRNA expression were enhanced in TSCs incubated with increasing concentrations of IGF-1 in the presence of BMP-2 (Fig. 4B and C). Adipogenic differentiation of TSCs in response to IGF-1+BMP-2 also occurred in a time-dependent manner (Fig. 4D, E and F). In order to further confirm the functions of endogenous IGF and BMP2, endogenous IGF-1 or BMP2 were knocked-down by shRNA, respectively. The BMP-2 and IGF-1 protein expression were significantly down-regulated by shRNA even in the presence of PGE2 (Fig. 5A). Moreover, BMP-2 shRNA or IGF-1 shRNA markedly reduced the adipocyte numbers (Fig. 5B and 5C), and also inhibited the PPARγ expression at the mRNA level (Fig. 5D). The results indicated that knock-down of either endogenous IGF-1 or BMP2 abolished PGE2-induced adipogenic differentiation.

### IGF-1 and BMP-2 mediate PGE2-induced adipogenic differentiation through activation of CREB and Smad

**Figure 6:**
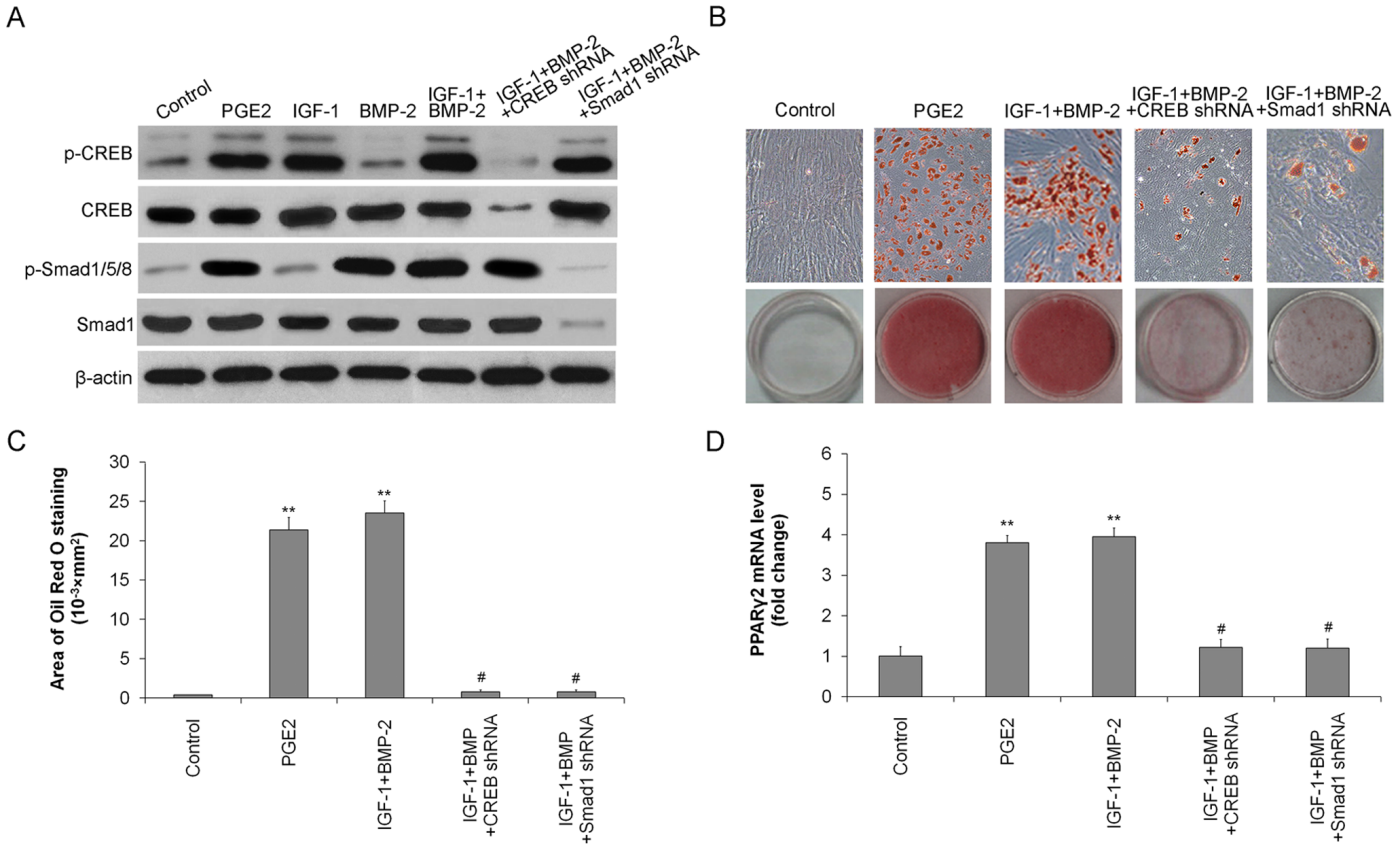
CREB and Smad are phosphorylated by IGF-1 and BMP-2, respectively, in PGE2-induced adipogenic differentiation. Rat TSCs were treated with PGE2 (100 ng/ml), IGF-1 (10 nM), BMP-2 (100 ng/ml), or IGF-1 (10 nM) + BMP-2 (100 ng/ml) with or without CREB shRNA or Smad shRNA. (A) Levels of total and phosphorylated CREB and Smad were determined by western blotting. β-actin was used as loading controls. TSCs were incubated with PGE2 (100 ng/ml), or IGF-1 (10 nM) + BMP-2 (100 ng/ml), with or without CREB shRNA or Smad shRNA. (B) Adipogenic differentiation detected by Oil Red O staining; (C) the Oil Red O staining areas in the cells were assessed; (D) PPARγ2 mRNA levels determined by qRT-PCR. The mRNA levels were normalized using GAPDH. Results represent the mean ± SD. **P<0.01 with respect to TSCs without PGE2; #P<0.05 with respect to TSCs with PGE2.

Given the critical roles of Smad in BMP2 signalling and CREB in IGF-1 signalling, we next examined whether activation of Smad and CREB is invovled the induction of PPARγ2 and adipocytic differentiation of TSCs. As shown in Fig. 6A, CREB and Smad were activated by phosphorylation in the presence of PGE2 or IGF-1+BMP-2. PGE2 or IGF-1, not BMP-2, increased the phosphorylation of CREB, and the effect of IGF-1 was blocked by CREB shRNA. Similarly, the phosphorylation of Smad was activated only by PGE2 or BMP-2, and was blocked by Smad1 shRNA (Fig. 6A). Either CREB shRNA or Smad shRNA markedly inhibited IGF-1+BMP-2-induced adipogenic differentiation, as indicated by Oil Red O Staining (Fig. 6B and 6C). CREB shRNA and Smad shRNA also inhibited expression of PPARγ2 at the mRNA level (Fig. 6D). These results indicated that IGF-1-activated pCREB, together with BMP2-activated pSmad, subsequently up-regulated the expression of PPARγ2, thus enhancing the adipogenic differentiation of TSCs.

## Discussion

The results of the current study confirmed that PGE2 induced the adipogenic differentiation of TSCs *in vitro.* Both IGF-1 and BMP-2 were implicated in the adipogenic differentiation of TSCs [24], [25], [26], and we also demonstrated that PGE2 induced IGF-1 gene and protein expression via cAMP/PKA/CEBPδ signalling pathway. However, neither IGF-1 nor BMP-2 alone was sufficient to induce adipogenic differentiation. Adipogenesis was significantly increased by treatment of TSCs with IGF-1 plus BMP-2. PGE2 also increased the phosphorylation of CREB and Smad via IGF-1 and BMP-2, respectively.

The degenerative changes seen in chronic tendinopathies are associated with mechanical stress, and the mechanisms responsible for chronic overuse tendon injuries may differ from those involved in acute tendon damage [29]. Although the role of inflammation in tendinopathies remains controversial, the inflammatory mediator PGE2 was increased in stretched tenocytes or tendons *in vitro* [15], [16], [17], [18], [19], [20], suggesting that it might be involved in the pathological changes associated with tendon overuse, including osteogenic and adipogenic changes. PGE2 was previously shown to induce BMP-2 [21], which in turn mediated osteogenic differentiation [23] and calcification [22]. The current study confirmed that PGE2 was also able to induce the adipogenic differentiation of TSCs.

BMPs are multifunctional growth factors with strong chondro-osteogenic effects. BMP-2 has been shown to mediate PGE2-induced osteogenic differentiation of human TSCs [23]. However, recent studies have shown that BMP-2 also exert adipogenic effects [30], [31], [32], and the BMP signalling pathway was required for commitment of C3H10T1/2 pluripotent stem cells to the adipocyte lineage [24]. It is possible that the involvements of BMP-2 in the osteogenic and adipogenic differentiation of TSCs are mediated by different BMP receptors [33], or may depend on BMP concentration [34], [35] and/or the presence of other intracellular and extracellular factors However, the results of the current study demonstrated that BMP-2 was necessary, but not sufficient, for inducing adipogenic differentiation of TSCs.

IGF-1 is also known to stimulate adipogenesis [25], [26]. IGF-1 attaches to its receptors and up-regulates phosphorylation of CREB via the PI3K/Akt pathway [25]. Activated CREB then increases the expression of PPARγ2, which acts as a crucial factor in adipogenic differentiation by controlling the expression of specific genes for adipocytes, such as phosphoenolpyruvate carboxykinase (PEPCK) and aP2 [25], [36]. This study showed that PGE2 stimulated IGF-1 synthesis via the cAMP/PKA/CEBPδ signalling pathway. However, as with BMP-2, IGF-1 alone was unable to induce PPARγ or adipogenic differentiation, and the presence of BMP-2 was also required. Knock-down of either IGF-1 or BMP2 abolished PGE2-induced adipogenic differentiation, further confirming the necessity of both IGF-1 and BMP2 in PGE2-induced adipogenic differentiation of TSCs.

Using shRNAs, we further demonstrated that CREB and Smad phosphorylation, induced by IGF-1 and BMP-2, respectively, were both required for induction of the adipogenic gene PPARγ2, confirming that the effects of PGE2 are transmited mainly via a CREB- and Smad-dependent mechanism.

In summary, the results of this study suggest that IGF-1 and BMP-2 together mediate PGE-2-induced adipogenic differentiation of TSCs. IGF-1 expression is up-regulated via the cAMP/PKA/CEBPδ pathway. IGF-1 and BMP2 phosphorylate and activate downstream CREB and Smad, respectively, which subsequently upregulate PPARγ2 expression, thus enhancing adipogenic differentiation. These findings provide the basis for further studies aimed at clarifying the pathogenesis of tendinopathies and identifying suitable therapeutic targets.
